# Japanese Encephalitis Virus Exploits Dopamine D2 Receptor-phospholipase C to Target Dopaminergic Human Neuronal Cells

**DOI:** 10.3389/fmicb.2017.00651

**Published:** 2017-04-11

**Authors:** Yogy Simanjuntak, Jian-Jong Liang, Yi-Ling Lee, Yi-Ling Lin

**Affiliations:** ^1^Institute of Biomedical Sciences, Academia SinicaTaipei, Taiwan; ^2^Genomic Research Center, Academia SinicaTaipei, Taiwan

**Keywords:** Japanese encephalitis virus, dopaminergic neuron, dopamine D2 receptor, phospholipase C, viral binding/entry molecule

## Abstract

Despite the availability of vaccines for Japanese encephalitis virus (JEV), the re-emerging virus remains a clinically important pathogen that causes acute encephalitis and permanent neuropsychiatric sequels. JEV highly targets dopaminergic neuron-rich brain regions including the thalamus and midbrain. The molecular mechanism contributing to the high susceptibility of these particular brain regions remains largely unclear. This study addressed whether this tissue tropism of JEV is associated with signaling of dopaminergic neurons. Three pieces of evidence indicate that JEV exploits dopamine signaling to facilitate its infection: (1) JEV infection modulates dopamine level; (2) a selective dopamine D2 receptor (D2R) agonist enhances JEV infection; and (3) stimulation of D2R activates phospholipase C (PLC) to enhance the surface expression of JEV binding/entry molecules, integrin β3 and vimentin. Overall, JEV may exploit dopamine-mediated neuronal communication to increase the susceptibility of D2R-expressing cells to JEV infection. This study identifies a potential underlying mechanism of viral invasiveness in the dopaminergic brain regions and suggests antiviral strategies against viral infection by targeting D2R-PLC signaling.

## Introduction

Japanese encephalitis virus (JEV), transmitted by mosquitoes, is the most clinically important etiological agent of viral encephalitis, with an estimated worldwide annual incidence of 30,000 to 50,000 cases. About 25–30% of these cases are fatal and 50% result in permanent neuropsychiatric sequel including impaired cognition, poliomyelitis-like paralysis and Parkinsonian movement disorders (Mackenzie et al., 2004). JEV, a flavivirus with positive-sense RNA genome, enters cell by receptor mediated endocytosis and the acidic pH of endosome causes conformational changes in envelope protein leading to release of genome into cytoplasm. Translation of viral RNA produces proteins required for viral RNA replication through RNA-dependent RNA polymerization along with host factors. Virus assembly is taken place within the endoplasmic reticulum-derived membrane compartments and non-infectious particles traverse through Golgi complex where furin-mediated cleavage of prM leads to conformational changes producing the infectious virion, which are then released into extracellular compartment through cellular secretory pathway. JEV invades the central nervous system (CNS) and primarily infects neuronal cells (Unni et al., 2011). In CNS infection, the thalamus and midbrain are the most severely affected (Kalita and Misra, 2000). Moreover, JEV RNA copy number is abundant in these brain areas as compared to other regions (Srivastava et al., 2013). However, we lack an understanding of the high susceptibility of these brain areas to JEV.

The midbrain is rich in number of dopaminergic neurons as the major source of dopamine in the mammalian CNS (Chinta and Andersen, 2005). In addition, the thalamus has been reported to be a key target for brain dopamine that also displays dopaminergic phenotypes (Sanchez-Gonzalez et al., 2005). Dopamine is the main catecholamine neurotransmitter that controls a wide variety of biological functions including voluntary movement, cognition, and endocrine regulation (Missale et al., 1998). Dopamine biosynthesis is regulated by phosphorylation of tyrosine hydroxylase (TH), the rate-limiting enzyme that hydrolyses tyrosine to L-DOPA (Daubner et al., 2011). The effects of dopamine are mediated by dopamine receptors (DRs), members of the G protein-coupled receptor (GPCR) superfamily. There are two types of DR: dopamine D1-like receptors including D1R and D5R subtypes and dopamine D2-like receptors including D2R, D3R, and D4R subtypes (Missale et al., 1998). Dopamine has greater binding affinity for D2- than D1-like receptors (Marcellino et al., 2012).

Signal transduction of DRs on agonist binding involves canonical cyclic adenosine monophosphate (cAMP) pathway. To produce cAMP from adenosine triphosphate (ATP), D1-like receptors activate adenylyl cyclase (AC) by inducing heterotrimeric G proteins Gαs and Gαolf. On the other hand, through Gαi and Gαo, D2-like receptors may inhibit the activation of AC (Beaulieu and Gainetdinov, 2011). Alternatively, D2R subtype and D2R-D1R heterodimer via Gαq and Gβγ can stimulate the release of intracellular calcium stores by activating inositol triphosphate receptor (IP_3_R) via inositol triphosphate (IP_3_), the product of phospholipase C (PLC) hydrolysis (Hernandez-Lopez et al., 2000; Yang et al., 2003; Lee et al., 2004).

Dopamine receptors are involved in virus infections. Dopamine can enhance HIV infection by up-regulating the surface HIV entry co-receptor in human macrophages and DR antagonists can inhibit this enhancement (Gaskill et al., 2014). Prochlorperazine, a D2R antagonist, can block Dengue virus (DENV) infection and protect mice against lethal DENV infection (Simanjuntak et al., 2015; Lai et al., 2017). Moreover, prochlorperazine also has an antiviral effect against JEV infection. However, whether JEV exploits dopaminergic signal transduction for viral replication is unknown. Here, we aimed to examine the interplay between JEV and dopaminergic neuronal cells and the underlying mechanism.

## Materials and Methods

### Viruses, Cell Lines, and Chemicals

The neurovirulent RP-9 strain of JEV was used for both *in vitro* and *in vivo* studies and was propagated in mosquito C6/36 cells as described (Chen et al., 1996). Viruses were titrated by plaque-forming assay in baby hamster kidney BHK-21 cells (ATCC: CCL-10). Dopaminergic human neuroblastoma BE(2)C cells (ATCC CRL-2268) were cultured in RPMI 1640 medium (Gibco) supplemented with 10% fetal bovine serum. Primary antibodies included anti-JEV-NS3, anti-phospho-tyrosine hydroxylase (Cell Signaling, #2791), anti-tyrosine hydroxylase (Cell Signaling, #2792), anti-D2R (Santa Cruz Biotechnology, sc-9113), anti-D1R (Santa Cruz Biotechnology, sc-1434), anti-phospho-CaMKII (Thermo Scientific, 22B1), anti-integrin β3 (BD Biosciences, 611140), anti-vimentin (Sigma, V6389), anti-β-actin (Chemicon), and anti-α-tubulin (Sigma–Aldrich).

### Mouse Study

Mouse experiments were approved and performed in accordance with the guidelines of the Academia Sinica Institutional Animal Care and Use Committee. Groups of 4-week-old AG129 mice were used to evaluate the effect of D2R agonist and antagonists on animal survival. Mice were challenged intraperitoneally with 5 × 10^4^ PFU/mouse of JEV and simultaneously injected with 30 μl PBS intracranially into the right hemisphere of the brain. Immediately, mice received PBS (vehicle control, *n* = 5) or 8 mg QH (D2R agonist)/kg body weight intravenously in the absence (QH, *n* = 5) or presence of either 8 mg D1R antagonist/kg body weight (QH+SCH23390, *n* = 5) or 8 mg D2R antagonist/kg body weight (QH+Haloperidol, *n* = 5). The animal survival was monitored daily.

### Virus Infection

Cells were adsorbed with JEV [multiplicity of infection (MOI), 1 or 5] for 2 h. Cells were washed to remove unbound virus and incubated for the indicated times. For antiviral assays, cells were adsorbed with JEV (MOI, 0.1) with the indicated doses of chemical compounds for 2 h, washed thoroughly, then incubated for 24 h with and without chemical compounds.

### Lentivirus Preparation and D2R-Knockdown

The lentivirus vector pLKO.1, which carries a short hairpin RNA (shRNA) targeting the human D2R (5′-GTCCTGGGAGACCCATGTAAA-3′, TRCN0000315421) or LacZ (5′-TGTTCGCATTATCCGAACCAT-3′, TRCN0000072223), obtained from the Taiwan National RNAi Core Facility, was cotransfected with pMD.G and pCMVΔR8.91 into HEK293T cells by use of Lipofectamine 2000 (Invitrogen). The lentiviruses were harvested from culture supernatants and concentrated by ultracentrifugation at 35,000 rpm in a Beckman SW41 rotor for 3.5 h at 4°C. The viral pellets were re-suspended and used to transduce BE(2)C cells. The D2R-deficient BE(2)C cells (shD2R-BE(2)C) and LacZ-control cells (shLacZ-BE(2)C) were selected with puromycin (10 μg/ml).

### Dopamine Assay

Quantitative assay of dopamine was performed according to the manufacturer’s protocol (Dopamine ELISA kit, KA3838-Abnova). Briefly, 250 μl of cell culture medium was used for enzymatic conversion. Sample absorbance was determined by use of an ELISA reader (Molecular Devices) at 450 nm.

### cAMP, Myo-inositol 1 Phosphate (IP_1_), and Calcium Measurements

cAMP level in cell lysate (10 μg) was measured by chemiluminescent enzyme-linked immunosorbent assay kit (Cell Biolabs). Quantitative assay of IP_1_ was performed according to the manufacturer’s protocol (IP-One Tb Cisbio Bioassays). Briefly, cells were plated in a 96-well black clear-bottom plate (50,000 cells/well) overnight. The level of IP_1_ in cell lysate was measured by use of SpectraMax M5 (Molecular Devices; Fluorescence ratio: 668/620 nm). Changes in intracellular cytosolic calcium level were evaluated by using calcium-sensitive dye Fluo-4AM (Calcium assay kit, Molecular Probes) with a fluorescence microplate reader (Spectramax, Molecular Devices). Cells were loaded with Fluo-4AM dye in HEPES-buffered Modified Hanks’ Balanced Salt Solution without phenol red and calcium. Cells were incubated for 10 min at 37°C. Quinpirole hydrochloride (QH) at the indicated doses was added to cells and fluorescence intensity was recorded at 5 min intervals over 30 min at room temperature.

### Preparation of Fluorescently Labeled JEV

Japanese encephalitis virus was purified and labeled with Lightning-Link Atto-488 (Innova Biosciences) as described (Liang et al., 2011). Briefly, virus was ultracentrifuged through a 35% sucrose cushion at 35,000 rpm in a Beckman SW41 rotor for 3.5 h at 4°C. The viral pellets were re-suspended in PBS and labeled with Lightning-Link Atto-488 (Innova Biosciences).

### Virus Binding/Entry Assays

Cells were adsorbed with JEV for 2 h with rocking on a linear shaker at room temperature for virus binding/entry assay. The cells were washed three times with cold PBS. Virus binding/entry was evaluated by confocal microscopy with fluorescently labeled (MOI, 20∼30) and plaque-forming assay with unlabeled JEV (MOI, 5). For virus binding assay, cells were adsorbed with JEV for 1 h at 4°C with rocking on linear shaker. For the virus entry assay, cells were adsorbed with JEV for 1 h at 4°C. After a gentle wash with cold medium, cells were shifted to 37°C incubator for 1 h to allow virus entry. Cells were then treated with acid glycine (0.1 M, pH = 3) to inactivate non-internalized virus for 5 min and washed with cold HBSS. Cells were harvested with use of cell scrapers, and the adsorbed viruses were released by passing the cells through a 27-G needle 3 times. Cell lysates were centrifuged at 12,000 rpm for 1 min, and the supernatant was used for plaque-forming assay.

### Flow Cytometry

Cells were plated in a 6-well plate for overnight. After treatments, cells were immunostained with anti-integrin β3 (BD Biosciences, 611140) or anti-vimentin (Sigma, V6389) without cell permeabilization for 1 h, then washed thoroughly and incubated with Alexa Fluor-488-conjugated secondary antibody (Molecular Probes) for 1 h at room temperature. After washing, cells were harvested with use of cell scrapers and passed through cell strainer. FACSCanto flow cytometry was used to measure signal intensity of respective proteins.

### Statistical Analysis

Data are presented as mean ± standard deviation (SD). For comparison of three or more categorical data, ANOVA-Bonferroni *post hoc* test was used. Two categorical data were compared by independent Student *t-*test. The statistical tests were two-tailed and significance was set at *P* < 0.05 and < 0.01. For immunoblotting, the band density was quantified by use of ImageJ (US National Institutes of Health). A survival curve was descriptively analyzed by use of SigmaPlot v10.0 (Systat Software). Determination of the median survival time (*T*_50_) and *P*-values, by the log-rank test, involved used of Prism v5.0 (GraphPad Software).

## Results

### JEV Modulates Dopamine Levels

**FIGURE 1:**
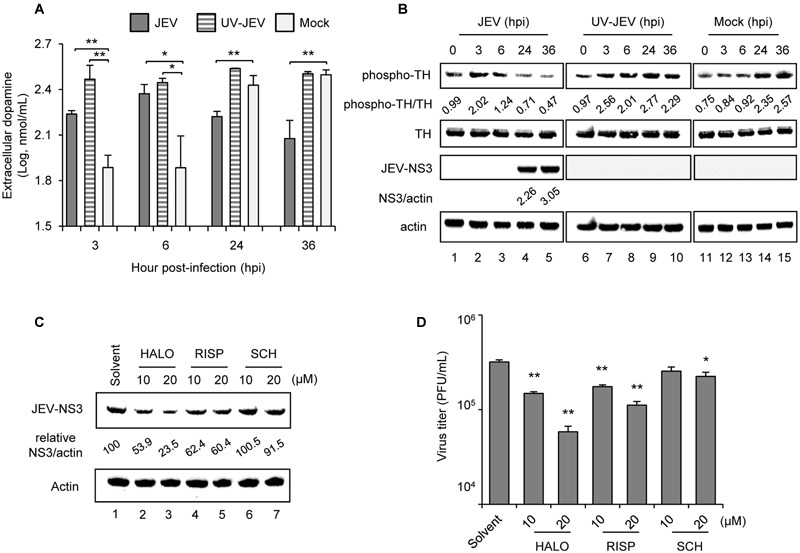
**Japanese encephalitis virus (JEV) modulates dopamine levels and D2R antagonists suppress JEV infection. (A,B)** Human BE(2)C cells were infected with JEV or UV-inactivated JEV for 3, 6, 24, and 36 h. ELISA of dopamine level in the culture supernatant **(A)**; and western blot analysis of protein levels of phospho-TH, total TH, JEV-NS3 and actin in cell lysates **(B)** at the indicated time [hours post-infection (hpi)]. Western blot analysis; relative ratio of phospho-TH to TH or NS3 to actin. **(C,D)** BE(2)C cells were infected with JEV with and without D2R antagonists [haloperidol (HALO) and risperidone (RISP)] or D1R antagonist SCH23390 (SCH) for 24 h. Western blot analysis of JEV-NS3 and actin; relative percentage of NS3 to actin was adjusted to solvent control **(C)**. Plaque-forming assay of viral progeny production in culture supernatant **(D)**. Data are mean ± SD (*n* = 3). ^∗^*P* < 0.05, ^∗∗^*P* < 0.01.

To investigate whether JEV affects dopamine production, we used dopaminergic human neuroblastoma BE(2)C cells, which show a high level of TH activity for dopamine synthesis (Kim et al., 1996). JEV-infected BE(2)C cells released a significantly higher level of dopamine at 3 and 6 h post-infection (hpi) as compared with mock-infected cells; however, at later times of 24 and 36 hpi dopamine release was reduced by JEV infection (**Figure 1A**), probably due to cytopathic effect caused by JEV. Consistently, JEV also modulated the rate-limiting enzyme of dopamine biosynthesis, with an increase of phospho-TH levels at 3 and 6 hpi and decrease at later times, when JEV-NS3 was abundant (**Figure 1B**). Notably, UV-inactivated JEV (UV-JEV) also significantly increased dopamine and phospho-TH levels at 3 and 6 hpi but different from that of JEV, the levels were not decreased by UV-JEV at 24 and 36 hpi (**Figures 1A,B**). These data suggest that JEV increases dopamine levels at the early stage of infection probably through viral binding/entry.

### D2R Antagonists Suppress JEV Infection

The biological effect of dopamine is mediated by DRs and to elucidate their role in JEV infection, we used selective antagonists targeting dopamine D1- or D2-like receptors (Missale et al., 1998). Similar to our previous study with D2R antagonist prochlorperazine (Simanjuntak et al., 2015), the D2R antagonists haloperidol and risperidone dose-dependently reduced JEV-NS3 protein expression and viral progeny production as compared with solvent-treated cells (**Figures 1C,D**). SCH23390, a D1R antagonist, also inhibited JEV infection, although it required a relatively high dose (**Figures 1C,D**). Thus, our data indicate the major role of D2R in JEV infection as compared with D1R.

### Stimulation of D2R Enhances JEV Infection

**FIGURE 2:**
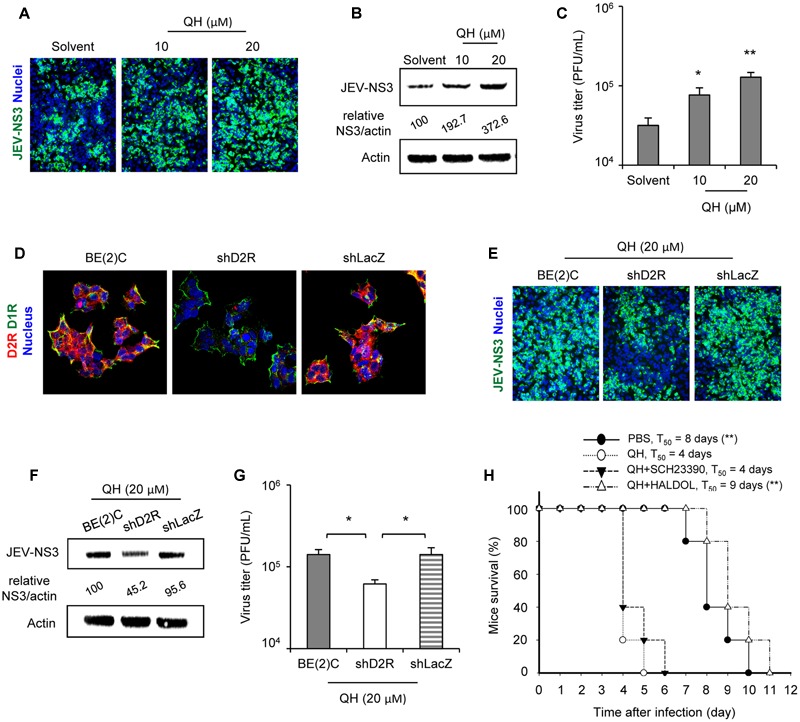
**Stimulation of D2R enhances JEV infection. (A–C)** BE(2)C cells were treated with the D2R-specific agonist quinpirole hydrochloride (QH) for 2 h, then infected with JEV for 24 h. Immunofluorescence microscopy (200× original magnification) of cells immunostained for JEV-NS3 (green) and Hoechst for nuclei (blue) **(A)**. Western blot analysis of JEV-NS3 and actin; relative percentage of NS3 to actin were adjusted to solvent control **(B)**. Plaque-forming assay of viral progeny production in culture supernatant **(C)**. **(D)** Knockdown efficiency of D2R expression in BE(2)C. Confocal microscopy (400× original magnification) of cells immunostained for D2R (red), D1R (green) and Hoechst for cell nuclei (blue). **(E–G)** Cells including BE(2)C, shD2R-BE(2)C, and shLacZ-BE(2)C were treated with QH for 2 h were infected with JEV for 24 h. Immunofluorescence microscopy (200× original magnification) of cells immunostained for JEV-NS3 (green) and Hoechst for nuclei (blue) **(E)**. Western blot analysis of JEV-NS3 and actin; relative percentage of NS3 to actin were adjusted to BE(2)C **(F)**. Plaque-forming assay of viral progeny production in culture supernatant **(G)**. Data are mean ± SD (*n* = 3). ^∗^*P* < 0.05, ^∗∗^*P* < 0.01. **(H)** AG129 mice were challenged with JEV and immediately received phosphate-buffered saline (PBS; *n* = 5) or 8 mg QH/kg body weight intravenously in the absence (QH, *n* = 5) or presence of either 8 mg D1R antagonist/kg body weight (QH+SCH23390, *n* = 5) or 8 mg D2R antagonist/kg body weight (QH+Haloperidol, *n* = 5). Mice survival was presented as percentage of survival. The median survival time (*T*_50_) and *P*-value, by the log-rank test, are presented. ^∗∗^*P* < 0.01, compared with QH-treated group.

To address the role of D2R activation in JEV infection, we used a D2R-specific agonist with putative dopaminergic properties, QH (Koller et al., 1987). Treatment of QH for 2 h before JEV infection dose-dependently enhanced JEV-NS3 expression and viral progeny production (**Figures 2A–C**). To verify the requirement of D2R in QH-enhanced JEV infection, we knocked down D2R expression in BE(2)C cells by transduction with a lentivirus expressing shRNA targeting human D2R (shD2R). The knockdown of D2R did not affect D1R expression and cellular localization (**Figure 2D**). The enhancement effect of QH on JEV-NS3 expression and viral progeny production was significantly lower in shD2R-BE(2)C cells than parental BE(2)C cells and knockdown control shLacZ-BE(2)C cells (**Figures 2E–G**). To further confirm the biological significance of D2R activation on JEV infection, we performed animal challenge experiment. QH treatment significantly accelerated animal mortality as compared with PBS treatment in JEV-challenged AG129 mice (*T*_50_: 4 days vs. 8 days) (**Figure 2H**). Notably, this acceleration of animal mortality was abolished in the presence of D2R antagonist (haloperidol; *T*_50_ = 9 days) but not D1R antagonist (SCH23390; *T*_50_ = 4 days) (**Figure 2H**). These data demonstrate that stimulation of D2R positively regulates JEV infection.

### JEV Infection and D2R Stimulation Activate Phospholipase C (PLC) Signaling Cascade

**FIGURE 3:**
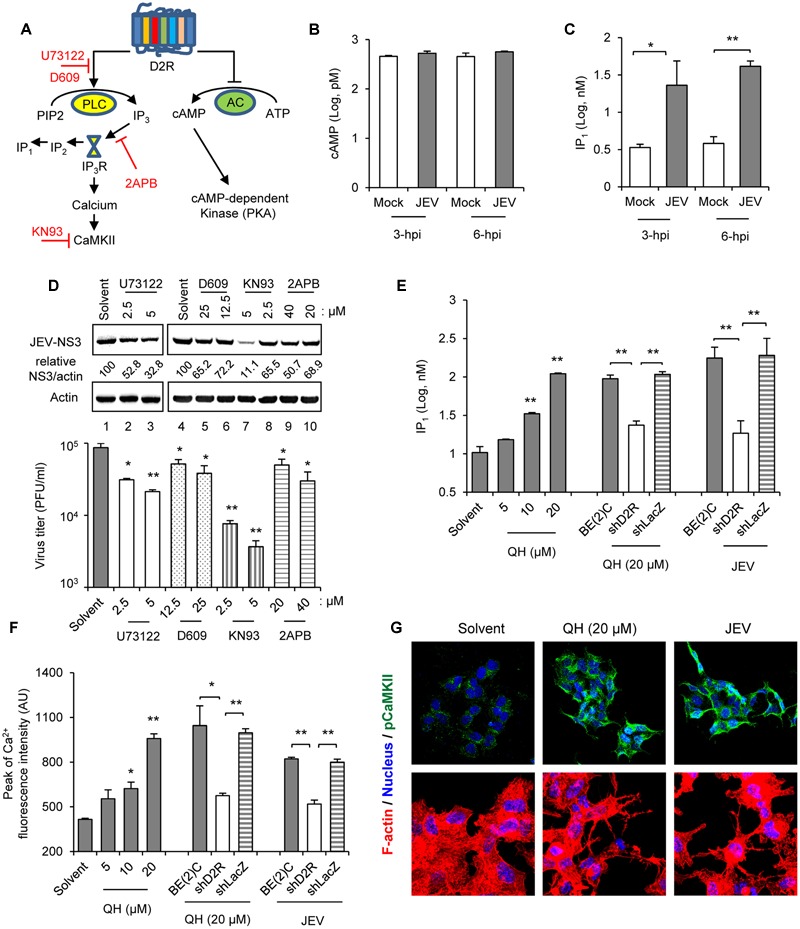
**Japanese encephalitis virus infection and D2R stimulation activate phospholipase C (PLC) signaling cascade. (A)** Major D2R signaling molecules (black) and the signaling inhibitors (red). **(B,C)** BE(2)C cells were infected with JEV for 3 and 6 h. Chemiluminescent ELISA of cAMP levels in the cell lysates **(B)**. IP_1_ levels in cell lysates were determined by homogeneous time-resolved fluorescence **(C)**. **(D)** BE(2)C cells were infected with JEV with and without PLC signaling cascade inhibitors at the indicated concentrations for 24 h. Western blot of JEV-NS3 and actin; relative percentage of JEV-NS3 to actin were adjusted to solvent control (upper panel). Plaque-forming assay of viral progeny production in culture supernatant (lower panel). **(E)** Cells including BE(2)C (gray), shD2R-BE(2)C (white), and shLacZ-BE(2)C (striped) were treated with D2R-specific agonist QH for 2 h at the indicated doses or infected with JEV for 3 h. IP_1_ levels in cell lysates were determined by homogeneous time-resolved fluorescence. **(F)** Cells were treated with QH for 30 min at the indicated doses or infected with JEV for 1 h, then the highest peak of Fluo-4 AM in the cells was determined by fluorescence microplate reader. **(G)** BE(2)C cells were treated with QH or infected with JEV for 5 h. Cells were immunostained for pCaMKII (green) and Hoechst for nuclei (blue). Confocal microscopy images are shown (400× original magnification, upper panel). Cells were stained with Alexa Fluor 568 phalloidin for F-actin (red) and Hoechst for nuclei (blue). Confocal microscopy images are shown (630× original magnification, lower panel). Data are mean ± SD (*n* = 3). ^∗^*P* < 0.05, ^∗∗^*P* < 0.01.

To determine the D2R signaling event involved in JEV life cycle, we evaluated the major pathways of AC and PLC downstream of D2R stimulation (**Figure 3A**). Through Gαi/αo, D2R may inhibit AC to negatively regulate cAMP level (Beaulieu and Gainetdinov, 2011), while via Gαq/βγ D2R may activate PLC to hydrolyzes phosphatidylinositol 4,5-biphosphate (PIP_2_) to inositol 1,4,5-triphosphate (IP_3_). IP_3_ then stimulates the release of calcium from intracellular stores before it is rapidly degraded to IP_2_ and then IP_1_ (Trinquet et al., 2006). At the early time of JEV infection when dopamine level was elevated (3∼6 h), cAMP levels were similar between mock and JEV-infected BE(2)C cells (**Figure 3B**). However, the levels of IP_1_ were significantly increased by JEV infection (**Figure 3C**). Furthermore, treatments with PLC signaling inhibitors at non-cytotoxic doses and in the range of their IC_50_ (Supplementary Figure S1) dose-dependently reduced JEV-NS3 expression and viral progeny production (**Figure 3D**). Thus, PLC signaling pathway is involved in JEV infection in dopaminergic BE(2)C cells.

Next, we verified the activation of PLC pathway by the D2R agonist QH. Treatment with QH dose-dependently increased IP_1_ production as compared with solvent treatment (**Figure 3E**). This QH-induced IP_1_ production was associated with D2R because IP_1_ level was lower in shD2R-BE(2)C than BE(2)C cells and knockdown control shLacZ-BE(2)C cells. Similarly, JEV infection induced IP_1_ production in a D2R-associated mechanism, since lower IP_1_ level was noted in cells with D2R knockdown (**Figure 3E**). In addition, QH dose-dependently increased cytosolic calcium level as measured by Fluo-4 AM calcium indicator assay (**Figure 3F**). Notably, cytosolic calcium level was lower in shD2R-BE(2)C than control cells with QH treatment, so QH-mediated calcium flux was associated with D2R. This D2R-dependent effect on cytosolic calcium level was also observed in JEV-infected cells (**Figure 3F**).

Calcium flux may activate calcium-dependent enzymes such as CaMKII, the most abundant neuronal protein kinase in the brain (Berridge et al., 2003; Okamoto et al., 2009). Calcium flux and CaMKII activation triggered by DR-mediated signaling can affect cell morphology by regulating cytoskeletal dynamics (Rosenberg and Spitzer, 2011). Treatment with QH and JEV infection for 5 h greatly increased phosphorylated CaMKII T286 (pCaMKII) level, a marker of CaMKII activation (Coultrap and Bayer, 2012), and also altered the cell shape to form neurite-like structures with high actin composition (**Figure 3G**). Together, these data demonstrate that D2R stimulation and JEV infection induces a PLC signaling cascade in BE(2)C cells.

### D2R-Mediated PLC Signaling Enhances JEV Binding/Entry

**FIGURE 4:**
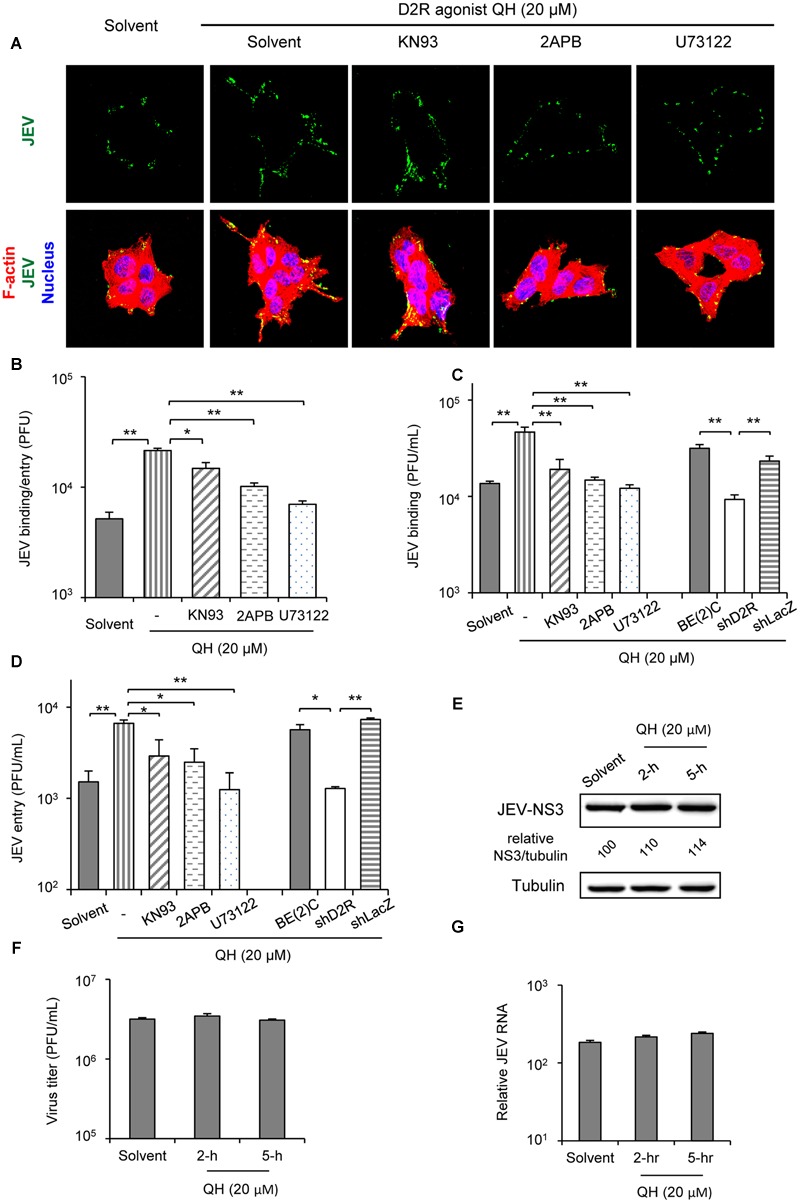
**D2R-mediated PLC signaling enhances JEV binding/entry. (A,B)** BE(2)C cells were treated with QH with and without PLC signaling inhibitors for 5 hr, then infected with fluorescently labeled JEV (**A**, green) or unlabeled JEV **(B)** for 2 h at room temperature with rocking on a linear shaker. Confocal microscopy (630× original magnification) of cells stained with Alexa Fluor 568 phalloidin for F-actin (red) and Hoechst for nuclei (blue) **(A)**. The level of virus binding/entry was quantified by plaque-forming assay **(B)**. **(C)** Cells were treated with QH with or without PLC signaling inhibitors for 5 h, then JEV binding was performed at 4°C for 1 h. The level of virus binding was quantified by plaque-forming assay. **(D)** Cells were treated with QH with or without PLC signaling inhibitors for 5 h, then cells were adsorbed with JEV at 4°C for 1 h. After washing with medium, cells were incubated at 37°C for an additional hour. The level of virus entry was quantified by plaque-forming assay. **(E–G)** BE(2)C cells were adsorbed with JEV for 2 h, then treated with QH (20 μM) for 2 or 5 h, replenished with QH-free medium, and incubated for 24 h. Western blot analysis of JEV-NS3 and actin; relative percentage of NS3 to tubulin were adjusted to solvent control **(E)**; plaque-forming assay of viral progeny production in culture supernatant **(F)**; and level of intracellular JEV RNA **(G)**. Data are mean ± SD (*n* = 3). ^∗^*P* < 0.05, ^∗∗^*P* < 0.01.

To dissect the viral life cycle affected by D2R-PLC activation, we performed viral binding/entry study in BE(2)C cells with or without QH pretreatment for 5 h. The amount of JEV binding/entry was significantly increased with QH treatment (**Figures 4A,B**). Furthermore, this enhancement in QH-treated cells was concomitantly reduced with PLC signaling cascade inhibitors KN93, 2APB, and U73122 (**Figures 4A,B**). To further address the effect on viral binding and/or viral entry, we performed virus binding and virus entry assays as described in the section “Materials and Methods.” Both JEV binding and entry were significantly increased by QH treatment and this enhancement could be repressed by PLC signaling inhibitors and with D2R knockdown in shD2R-BE(2)C cells (**Figures 4C,D**). However, D2R stimulation had no effect on post-entry step of JEV infection because treatments with QH after virus adsorption did not significantly change JEV-NS3 expression, virus progeny production, and intracellular JEV RNA level (**Figures 4E–G**). Therefore, D2R-mediated PLC signaling plays a role in enhancing JEV binding/entry.

### D2R-PLC Activation Increases the Surface Expression of JEV Binding/Entry Molecules

**FIGURE 5:**
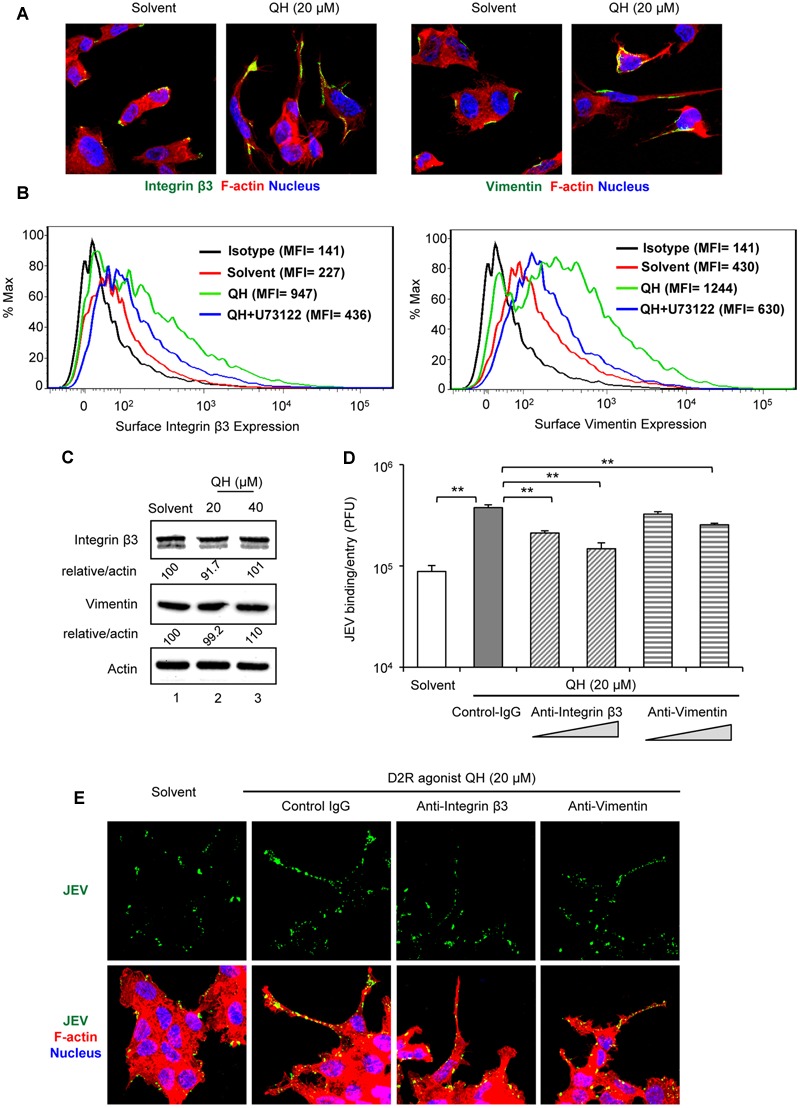
**D2R-PLC activation increases the surface expression of JEV binding/entry molecules.** BE(2)C cells were treated with or without D2R-specific agonist QH at the indicated concentration for 5 h. **(A,B)** Without permeabilization, cells were immunostained for integrin β3 [green, (**A**, left 2 panels)] or vimentin [green, (**A**, right 2 panels)]. Cells were also stained with Alexa Fluor 568 phalloidin for F-actin (red) and Hoechst for nuclei (blue). Confocal microscopy images (630× original magnification). Histogram [mean fluorescence intensity (MFI)] of surface integrin β3 (**B**, left panel) or vimentin (**B**, right panel) of QH-treated cells with or without specific PLC inhibitor (U73122, 5 μM) was measured by flow cytometry. **(C)** Western blot analysis of integrin β3, vimentin and actin; relative percentage of integrin β3 or vimentin to actin were adjusted to solvent control. **(D,E)** BE(2)C cells were infected with unlabeled JEV **(D)** or fluorescently labeled JEV (**E**, green) for 2 h at room temperature with or without anti-integrin β3 and anti-vimentin antibody (0.75 and 1.5 μg/ ml). Mouse immunoglobulin G (IgG) was used as antibody control (1.5 μg/ml). The level of virus binding/entry was quantified by plaque-forming assay **(D)**. Cells with or without antibody (1.5 μg/ ml) were stained with Alexa Fluor 568 phalloidin for F-actin (red) and Hoechst for nuclei (blue). Confocal microscopy images (630× original magnification) **(E)**. Data are mean ± SD (*n* = 3). ^∗∗^*P* < 0.01.

Phospholipase C activation increases cytosolic calcium level, which in turn activates calcium-dependent kinases and/or directly enhances actin assembly to facilitate the formation of focal adhesion on the cell surface (Prevarskaya et al., 2011). One of the main components of focal adhesion, αvβ3 integrin adhesion receptor, has been reported to mediate entry of flavivirus such as West Nile virus and JEV (Chu and Ng, 2004). Furthermore, integrin β3 is involved in recruiting vimentin to the cell surface (Bhattacharya et al., 2008) and our previous work indicated the role of vimentin in JEV binding and virulence (Liang et al., 2011). Thus, we tested whether the surface expression of integrin β3 and vimentin was increased with treatment of the D2R agonist, QH. Treatment with QH for 5 h greatly increased the surface expression of integrin β3 and vimentin as compared to solvent-treatment in BE(2)C cells (**Figures 5A,B**) dependent on PLC because the PLC inhibitor U73122 reversed this effect (**Figure 5B**). The increased surface expression of integrin β3 and vimentin was not likely due to the effect on protein expression because their total protein levels were not changed by QH (**Figure 5C**).

To support the role of integrin β3 and vimentin in D2R-PLC-enhanced JEV binding/entry, we performed antibody-blockage experiments. BE(2)C cells-treated with QH for 5 h underwent viral binding/entry assay with and without antibodies against integrin β3 and vimentin. The enhanced JEV binding/entry with QH was significantly repressed by anti-integrin β3 and anti-vimentin antibodies as compared with mouse IgG control (**Figures 5D,E**). These data suggest that D2R-PLC-enhanced JEV binding/entry is associated with the increased surface expression of JEV binding/entry molecules including integrin β3 and vimentin.

## Discussion

**FIGURE 6:**
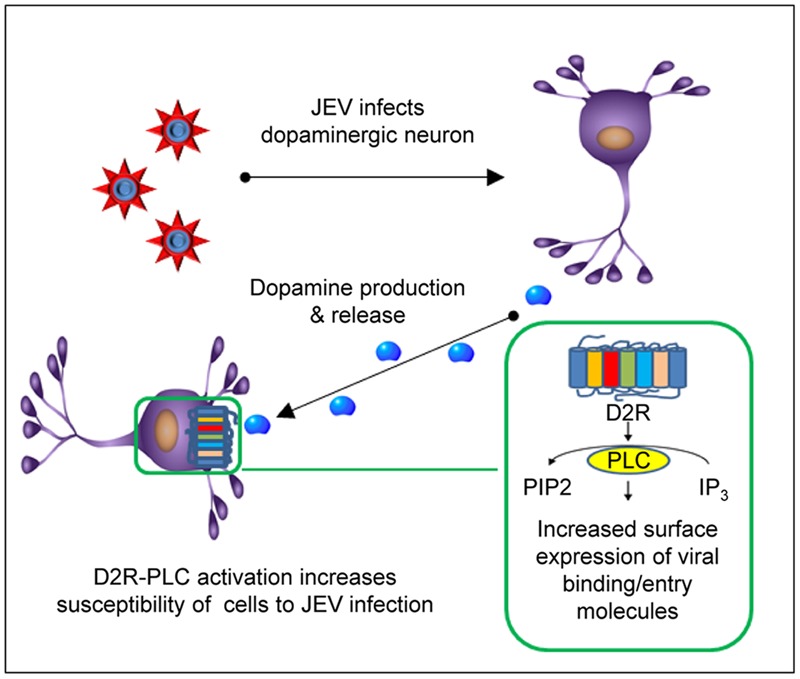
**Japanese encephalitis virus infection in dopaminergic neurons.** JEV infects dopaminergic neurons and upregulates dopamine production and release at the early stage of infection. This in turn may activate D2R-PLC signaling pathway of the surrounding D2R-expressing cells. Activation of D2R-PLC increases surface expression of the molecules involved in JEV binding/entry and then enhances JEV infection.

Japanese encephalitis virus infection of the brain mostly targets dopaminergic neuron-rich areas, the thalamus and midbrain (Kalita and Misra, 2000; Chinta and Andersen, 2005; Sanchez-Gonzalez et al., 2005; Srivastava et al., 2013). Here, we provide evidence to explain this tissue tropism by revealing an interesting approach adapted by JEV to exploit the dopaminergic system to enhance its viral life cycle. The dopamine production is significantly increased at the early stage of JEV infection. The increased level of dopamine may be implicated in the susceptibility of neighboring cells to JEV via D2R-PLC-associated signal transduction. Stimulation of D2R by dopamine activates PLC signaling cascade. PLC hydrolyzes phosphatidylinositol 4,5-biphosphate (PIP_2_) to inositol 1,4,5-triphosphate (IP_3_) (Trinquet et al., 2006). IP_3_ then stimulates the release of calcium from intracellular stores which in turn activate calcium-dependent enzymes such as CaMKII, the most abundant neuronal protein kinase in the brain (Berridge et al., 2003; Okamoto et al., 2009). CaMKII activates focal adhesion kinase to mediate formation of surface focal adhesion including αvβ3 integrin that plays a role in JEV entry (Chu and Ng, 2004; Prevarskaya et al., 2011). Furthermore, integrin β3 is involved in the cell surface recruitment of vimentin, a binding molecule of JEV in neuronal cells (Bhattacharya et al., 2008; Liang et al., 2011). Thus, D2R-PLC activation may increase JEV binding/entry to enhance JEV infection (as outlined in **Figure 6**).

Our data indicate that JEV can induce hyperdopaminergia during the early stage of infection. Presumably, elevated dopamine level above the basal level may confer favorable conditions for viral life cycle, because hyperdopaminergia may alter cellular responses such as protein kinase C-mediated ERK and Akt expression (Beaulieu and Gainetdinov, 2011). Involvement of hyperdopaminergia is implied in HIV infection. Methamphetamine, a psychostimulant that increases the level of extracellular dopamine, enhances HIV infection in human macrophages (Liang et al., 2008; Taylor et al., 2013). Moreover, treatment with dopamine significantly increases HIV entry by enhancing the surface expression of CCR5 via DR-PLC activation (Gaskill et al., 2014). Several meningoencephalitis viruses have been reported to activate PLC. For example, M33, a GPCR encoded by murine cytomegalovirus, can trigger protein kinase C signaling via Gq protein-coupled PLC activation to support viral replication and take a part in pathogenesis (Sherrill et al., 2009). Group B coxsackieviruses, a leading cause of aseptic meningitis, can induce PLC-dependent intracellular calcium release to activate a cysteine protease for entering brain endothelial cells (Bozym et al., 2010). In addition, PLC signaling plays a role in the entry of influenza A virus H1N1 but not H3N2, via an unclear mechanism (Zhu et al., 2014). Interestingly, genome wide association studies (GWAS) of dengue patients revealed a single nucleotide polymorphism (SNP) in the gene PLC, epsilon 1, associated with severe dengue shock syndrome (Khor et al., 2011; Dang et al., 2014). Here, we add JEV to the list of viruses whose life cycle interacts with the D2R-PLC pathway.

Emerging and clinically important flaviviruses have ability to enter the CNS and infect neuronal cells (Sips et al., 2012). For instance, West Nile virus and dengue virus infections cause pathological features in the thalamus, another main source of brain dopamine (Solomon, 2004; Sanchez-Gonzalez et al., 2005; Malik et al., 2014). Zika virus (ZIKV), a recent public health problem, has been linked to neonatal microcephaly in that ZIKV can target developing brain cells (Garcez et al., 2016). Interestingly, dopamine and D2R may play a critical role in the developing human brain (Rothmond et al., 2012). Whether dopamine-related signaling may also take part in the life cycle and pathogenesis of these flaviviruses remains elusive.

Overall, our results revealed elevated dopamine level at the early stage of JEV infection to enhance viral entry in D2R-expressing cells via a PLC-mediated signaling pathway. Our study proposes a possible explanation for the high susceptibility of dopaminergic brain regions to viral pathogenesis and suggests a therapeutic strategy against viral infection by targeting D2R-PLC signal transduction.

## Author Contributions

YS and Y-LLi designed research, analyzed data and wrote the paper. YS performed research. YS and J-JL performed animal study. Y-LLe provided critical reagents.

## Conflict of Interest Statement

The authors declare that the research was conducted in the absence of any commercial or financial relationships that could be construed as a potential conflict of interest.
