# Supplementary material for: Japanese Encephalitis Virus Exploits Dopamine D2 Receptor-phospholipase C to Target Dopaminergic Human Neuronal Cells
Source: Front Microbiol. 2017 Apr 11;8:651. doi: 10.3389/fmicb.2017.00651 (PMC5387065; doi:10.3389/fmicb.2017.00651)
Supplement: Supplementary file 1 [file Image_1.PDF]

Japanese Encephalitis Virus Exploits Dopamine D2 Receptor-Phospholipase C to Target Dopaminergic Human Neuronal Cells

Yogy Simanjuntak[1*], Jian-Jong Liang[1],Yi-Ling Lee[1], Yi-Ling Lin[1,2,*]

[1]Institute of Biomedical Sciences, and [2]Genomic Research Center, Academia Sinica, Taipei, Taiwan

Keywords: Japanese encephalitis virus; dopaminergic neuron; dopamine D2 receptor; phospholipase C; viral binding/entry molecule

Running title: JEV infection in dopaminergic neuron

[*]**Correspondence:**
Yogy Simanjuntak, Ph.D., E-mail: yogy@ibms.sinica.edu.tw
Yi-Ling Lin, Ph.D., E-mail: yll@ibms.sinica.edu.tw

Mailing Address: Institute of Biomedical Sciences, Academia Sinica, No.128, Sec. 2, Academia Road, Taipei 11529, Taiwan.
Telephone: (886)-2-2652-3902; **Fax:** (886)-2-2785-8847

Supplemental Figure S1. Cytotoxicity assays of drugs and inhibitors. (A and B) Cytotoxicity assays of drugs and inhibitors. BE(2)C cells were treated with DMSO (solvent) or the indicated concentrations of chemicals for 24 hr. Lactate dehydrogenase (A) and XTT (B) assays were performed to determine cytotoxicity and cell proliferation, respectively. Data are mean ± SD (n = 3). (C) Function and half maximal inhibitory concentration (IC_{50}) of the signaling inhibitors.

A

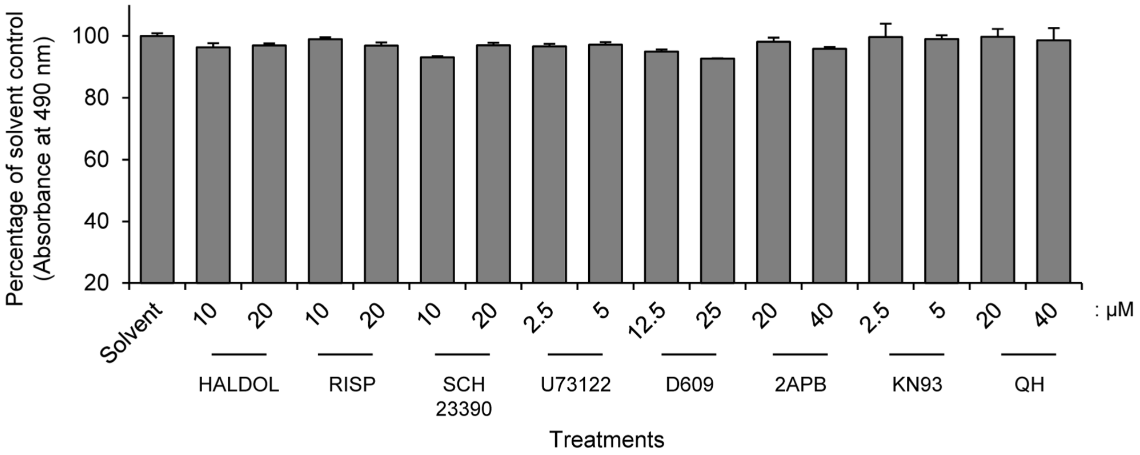

B

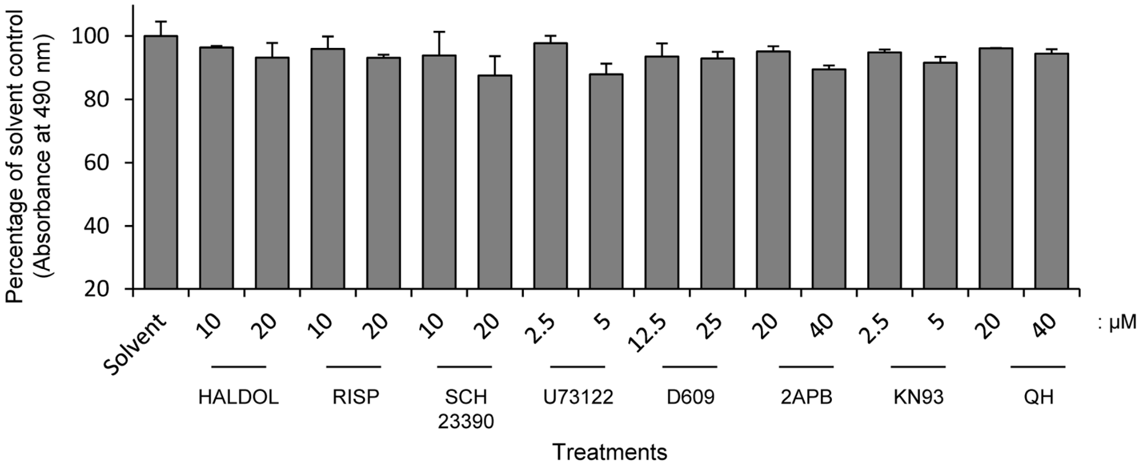

C

Function	Inhibitor	IC_{50} (µM)	References
Inhibitor of Phospholipase C (PLC)	U-73122	2-10	Santa Cruz Biotech (CAS 112648-68-7) http://www.selleckchem.com/products/u73122.html
	D609	94	Santa Cruz Biotech (CAS 83373-60-8)
Inhibitor of inositol triphosphate receptor (IP_3R)	2-APB	42	Santa Cruz Biotech (CAS 524-95-8)
Inhibitor of calcium/calmadulin-dependent kinase II (CaMKII)	KN-93	0.3-4	Santa Cruz Biotech (CAS 139298-40-1) Pellicena, P. J. & Schulman, H. CaMKII inhibitors: from research tools to therapeutic agents. *Front Pharmacol* 5 (21), 1-10 (2014)
